# Blockade of Platelet Glycoprotein Ibα Augments Neuroprotection in Orai2-Deficient Mice during Middle Cerebral Artery Occlusion

**DOI:** 10.3390/ijms23169496

**Published:** 2022-08-22

**Authors:** Michael Bieber, Michael K. Schuhmann, Maximilian Bellut, David Stegner, Katrin G. Heinze, Mirko Pham, Bernhard Nieswandt, Guido Stoll

**Affiliations:** 1 Department of Neurology, University Hospital Wuerzburg, 97080 Wuerzburg, Germany; 2 Rudolf Virchow Center for Integrative and Translational Bioimaging, University of Wuerzburg, 97080 Wuerzburg, Germany; 3 Institute of Experimental Biomedicine, University Hospital Wuerzburg, 97080 Wuerzburg, Germany; 4 Department of Neuroradiology, University Hospital Wuerzburg, 97080 Wuerzburg, Germany

**Keywords:** ischemic penumbra, Orai2, glycoprotein receptor Ibα, ischemic stroke, thrombo-inflammation, middle cerebral artery occlusion

## Abstract

During ischemic stroke, infarct growth before recanalization diminishes functional outcome. Hence, adjunct treatment options to protect the ischemic penumbra before recanalization are eagerly awaited. In experimental stroke targeting two different pathways conferred protection from penumbral tissue loss: (1) enhancement of hypoxic tolerance of neurons by deletion of the calcium channel subunit Orai2 and (2) blocking of detrimental lymphocyte–platelet responses. However, until now, no preclinical stroke study has assessed the potential of combining neuroprotective with anti-thrombo-inflammatory interventions to augment therapeutic effects. We induced focal cerebral ischemia in Orai2-deficient (*Orai2^-/-^*) mice by middle cerebral artery occlusion (MCAO). Animals were treated with anti-glycoprotein Ib alpha (GPIbα) Fab fragments (p0p/B Fab) blocking GPIbα–von Willebrand factor (vWF) interactions. Rat immunoglobulin G (IgG) Fab was used as the control treatment. The extent of infarct growth before recanalization was assessed at 4 h after MCAO. Moreover, infarct volumes were determined 6 h after recanalization (occlusion time: 4 h). Orai2 deficiency significantly halted cerebral infarct progression under occlusion. Inhibition of platelet GPIbα further reduced primary infarct growth in *Orai2^-/-^* mice. During ischemia–reperfusion, upon recanalization, mice were likewise protected. All in all, we show that neuroprotection in *Orai2^-/-^* mice can be augmented by targeting thrombo-inflammation. This supports the clinical development of combined neuroprotective/anti-platelet strategies in hyper-acute stroke.

## 1. Introduction

For acute stroke patients with large vessel occlusion (LVO) the advent of mechanical thrombectomy (MT) dramatically improved outcomes. However, even with successful recanalization, up to 50% of patients are still left with significant disability or die. In particular, primary infarct growth, i.e., early infarct growth before recanalization, has a strong impact on the treatment success of MT [1]. Recently, two principal means have emerged to limit progressive infarction before recanalization: (1) targeting platelet driven intravascular thrombo-inflammation and (2) classical neuronal protection [2,3,4]. We could show that targeting the early steps of platelet adherence to vessel walls via glycoprotein (GP) Ibα–von Willebrand factor (vWF) interactions can delay progressive brain infarctions by blocking platelet–lymphocyte responses [4,5,6]. In addition, we found a critical contribution of the Ca^2+^ channel Orai2 to neuronal cell death following hypoxia by triggering excessive cytosolic Ca^2+^ accumulation. Mice lacking Orai2 displayed reduced cerebral damage both during acute ischemia under vessel occlusion and during ischemia–reperfusion upon recanalization [7]. 

In the present study, we examined whether neuroprotection in Orai2-deficient (*Orai2^-^^/-^*) mice under LVO could be augmented by combined blockade of platelet GPIbα in mice in delaying infarct progression before recanalization. 

## 2. Results

**Figure 1:**
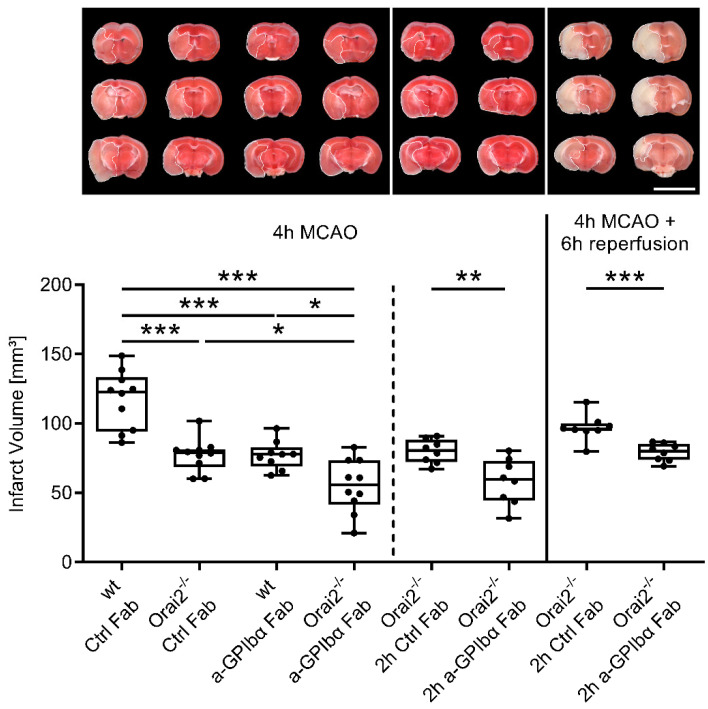
Combining Orai2 deficiency with blocking of platelet GPIbα additively delays ischemic brain damage. Representative images of coronal brain sections stained with TTC, 4 h after MCAO or after 4 h of MCAO, with additional 6 h of reperfusion in mice treated with rat IgG Fab (Ctrl Fab) or p0p/B Fab (a-GPIbα Fab) immediately or 2 h after MCA occlusion. Infarcted areas are shown in white. Scale bar = 10 mm. Planimetric analyses were used to quantify the infarct volumes. Results are presented as box plots (n = 8–10). * *p* < 0.05, ** *p* < 0.01, *** *p* < 0.001 between the indicated groups. MCAO, middle cerebral artery occlusion.

First, we occluded the middle cerebral artery (MCA) of *Orai2^-/-^* and wild-type (wt) mice for 4 h. Mice were treated immediately with control Fab fragments upon vessel occlusion. Strikingly, infarct volumes in *Orai2^-/-^* mice were significantly reduced compared to wt mice (~36%), as revealed by TTC staining (Figure 1), which means that infarct growth under occlusion before recanalization is targetable. In addition, infarct volumes in wt mice treated with anti-GPIbα Fab fragments targeting thrombo-inflammation also showed a significant reduction compared to wt mice (~37%). Most importantly, treatment of *Orai2^-/-^* mice with anti-GPIbα Fab fragments augmented the protective effect of Orai2 deficiency, since the degree of protection exceeded the effects in control Fab fragment-treated *Orai2^-/-^* mice or anti-GPIbα Fab fragment-treated wt mice, respectively (~30%).

To prove that the apparent protective effect of targeting platelet GPIbα in *Orai2^-/-^* mice is also effective with delayed treatment, next, we administered the anti-GPIbα Fab fragment 2 h after vessel occlusion. Again, a stroke-mitigating effect was seen at 4 h after MCA occlusion (~26%). 

In addition, reduced infarct growth under occlusion (~17%) persisted into the reperfusion phase in *Orai2^-/-^* mice, when the primary occlusion phase of 4 h was followed by 6 h of reflow and the animals received a delayed treatment with anti-GPIbα Fab fragments 2 h after MCA occlusion.

**Figure 2:**
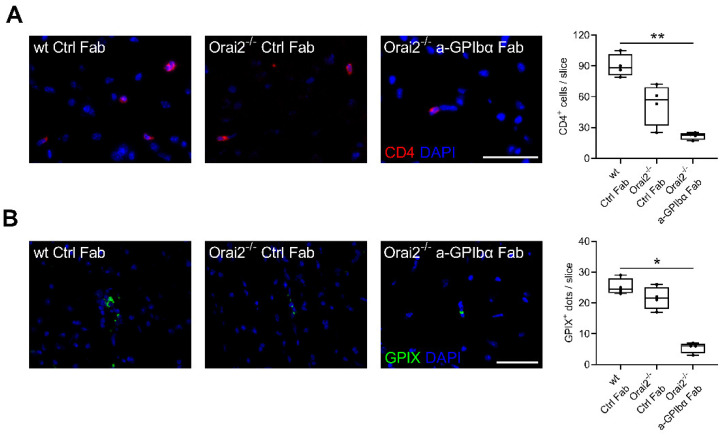
Blocking of platelet GPIbα diminished ultra-early T-cell recruitment in the ischemic brain of Orai2-deficient mice. (**A**) Representative immunocytologic stainings (left) and quantification (right) of brain-infiltrating CD4-positive T-lymphocytes (Cy3, red) and nuclei (DAPI, blue) in the whole ipsilateral hemisphere 4 h after MCAO in mice treated with rat IgG Fab (Ctrl Fab) or p0p/B Fab (a-GPIbα Fab) immediately after MCA occlusion, using a 40x objective lens. Scale bar = 50 µm (n = 4). (**B**) Representative immunocytologic stainings (left) and quantification (right) of ipsilesional glycoprotein IX (GPIX)-positive aggregates (Alexa 488, green) and nuclei (DAPI, blue) in the whole ipsilateral hemisphere 4 h after MCAO in mice treated with rat IgG Fab (Ctrl Fab) or p0p/B Fab (a-GPIbα Fab) immediately after MCA occlusion, using a 40x objective lens (n = 4). * *p* < 0.05, ** *p* < 0.01 between the indicated groups. MCAO, middle cerebral artery occlusion.

Next, we assessed the effect of targeting platelet GPIb on the infiltration of T-cells and platelet aggregation under MCA occlusion in Orai2-deficient mice. Similar to previous studies [4,6], 4 h after MCA occlusion, anti-GPIbα treatment reduced the number of infiltrated T-lymphocytes as well as platelet aggregates in the ipsilesional hemisphere of *Orai2^-/-^* mice (Figure 2).

## 3. Discussion

As our principal finding, we show that neuronal protection from ischemic/hypoxic cell death in Orai-2-deficient mice can be augmented in mice by targeting thrombo-inflammation under occlusion. 

It is well established that if, under LVO, timely recanalization cannot be achieved, infarcts rapidly grow and that the velocity of penumbral tissue loss depends on the degree of collateral blood flow [1]. To date, typical patients eligible for MT have to be transferred frequently from remote hospitals to primary stroke centers, causing significant delays which lead to clinically highly relevant early infarct growth, which in turn is closely associated with loss of favorable outcomes [8]. This current core clinical problem makes urgent experimental and clinical studies aiming to prevent infarct growth before recanalization and ischemia–reperfusion injury thereafter. In this context, the concept of neuroprotection may experience a renaissance [9]. Recently, an ongoing clinical approach (ESCAPE-NA1) has heightened optimism that neuroprotective strategies in human stroke are feasible when combined with MT [2]. Important for the success of any neuroprotective intervention during LVO is residual blood flow from macrovascular collateral anastomoses, which on the one hand prevents immediate cell death [10] and on the other hand represents the entry path for intravenously administered drugs to reach the target territory, despite total embolic occlusion [11]. We have previously shown that Orai2 is crucial for ischemic neuronal cell death and that its absence can substantially delay progressive brain infarction before recanalization [7]. Similarly, blocking of detrimental leukocyte- and GPIbα-mediated responses also diminished primary infarct growth during middle cerebral artery occlusion in mice [4], indicating that similar thrombo-inflammatory mechanisms to those identified in ischemia–reperfusion injury, in which platelet and T-cell interactions cause infarct growth in acute stroke [3], are set in motion during primary macrovascular occlusion. We now extend these studies by showing, for the first time, that neuroprotection in *Orai2^-/-^* mice can be augmented by anti-platelet treatment targeting thrombo-inflammation. The fact that blocking of platelet GPIbα diminished T-cell recruitment in the ischemic brain of Orai2-deficient mice provides further evidence that T-cells interact with platelets and facilitate infarct growth under occlusion. These experimental data support the clinical development of combined neuroprotective/anti-platelet strategies in hyper-acute stroke.

One limitation of our proof-of-concept study was the inclusion of only young mice to limit the variability of the MCAO model. Since stroke patients are typically elderly and suffer from comorbidities, an extension of the study’s findings in old mice with additional cardiovascular diseases will be necessary. Another limitation was the inclusion of male mice only. We are aware that sex-related differences can have an impact on stroke outcomes [12]. Therefore, in order to fully establish a treatment method for immediate clinical translation, more research is required.

## 4. Materials and Methods

### 4.1. Animals

We randomized male *Orai2^-/-^* mice and wild-type littermates (6–8 weeks old) and subjected them to a MCAO [4]. Animal studies were approved by the district government of lower Franconia and were conducted in accordance with the US National Institutes of Health Guide for the Care and Use of Laboratory Animals. The experiments were designed, performed and reported according to the Animal Research: Reporting of In Vivo Experiments guidelines [13]. 

### 4.2. Ischemia Model

Focal cerebral ischemia was induced by a 4 h MCAO or a 4 h MCAO with a 6 h reperfusion phase [4]. Occlusion times of 4 h were chosen to reflect the mean time from symptom onset to recanalization of 285 (210–362) min as revealed in a meta-analysis of 5 randomized trials of endovascular thrombectomy after ischemic stroke due to large vascular occlusion [14]. Mice for all animal experiments were randomized and coded by an independent researcher who was not involved in the data analysis, so experiments were carried out blindly. Investigators involved in the surgery and evaluation of all readout parameters were blinded to the experimental groups. To reduce the variability of our outcome parameters caused by sex differences and thereby to decrease group sizes, we used only male mice in the study. In recent studies, severe effects of sex differences on infarct sizes and inflammatory response were found [12,15]. Mice were excluded from endpoint analyses for the following pre-specified reasons: (1) death before the predefined experimental endpoint; (2) dropout score (weight loss, general condition, spontaneous behavior); (3) operation time > 10 min (to exclude the influence of prolonged anesthesia and increase group comparability). For induction of MCAO, mice were anesthetized with 2% isoflurane in O_2_ (*v/v*) and subcutaneously injected with 200 mg/kg of body weight of Metamizol. Lidocaine gel was used on the margin of the wound as an analgesia. To maintain core body temperature close to 37 °C throughout surgery, a servo-controlled heating blanket was used. After a midline neck incision, a standardized silicon rubber-coated no. 6.0 nylon monofilament (6023910PK10; Doccol, Sharon, MA, USA) was inserted into the right common carotid artery and advanced via the internal carotid artery to occlude the origin of the MCA for 4 h. For the 4 h MCAO/6 h reperfusion group, after 4 h, mice were re-anesthetized and the occluding filament was removed to allow reperfusion. Sample size calculation was performed using estimates of the typical experimental brain infarct volume from previous studies [4,6], a standard deviation of 20% to the respective mean values, a power of 90% and a probability of a type I error of <5%. Therefore, a group size of ≥8 was necessary to confidently detect a difference of 30% in stroke size.

### 4.3. Triphenyltetrazolium Chloride (TTC) Staining

Animals were sacrificed 4 h after MCAO or after 4 h MCAO with 6 h reperfusion phase and the brains were cut into three 2 mm-thick coronal sections. The slices were stained for 20 min at 37 °C with 2% TTC to visualize the infarctions. Edema-corrected infarct volumes were calculated by planimetry (ImageJ software version 1.53q, National Institutes of Health, Bethesda, MD, USA) [4].

### 4.4. Animal Treatment 

Mice received 100 μg p0p/B antigen-binding fragment (Fab) i.v. immediately or 2 h after stroke induction to inhibit platelet GPIbα. Controls received 100 μg rat IgG Fab [6].

### 4.5. Immunohistochemistry

For immunohistochemistry, mouse brain sections were fixed with methanol and blocked with 10% BSA. Staining was performed with antibodies against CD4 (BioLegend, #100506; dilution 1:50) and GPIX (emfret; dilution 1:100), as described previously [4,16].

### 4.6. Statistical Analyses

All data are presented as box plots, including medians (Med) with the 25th percentile (25%), the 75th percentile (75%), minimum and maximum. For statistical analysis, the GraphPad Prism 9 software package was used. Data were tested for Gaussian distribution with the D’Agostino–Pearson omnibus normality test and then analyzed by one-way analysis of variance (ANOVA) with post hoc Bonferroni adjustment for *p*-values or, for nonparametric analysis, compared using the Kruskal–Wallis test with post hoc Dunn’s multiple comparison test. If only 2 groups were compared, an unpaired, two-tailed Student’s *t*-test was applied. Probability values <0.05 were considered to indicate statistically significant results [4]

## Data Availability

The analyzed data sets generated during the study are available from the corresponding author on reasonable request.
